# A dynamic time order network for time-series gene expression data analysis

**DOI:** 10.1186/1752-0509-6-S3-S9

**Published:** 2012-12-17

**Authors:** Pengyue Zhang, Raphaël Mourad, Yang Xiang, Kun Huang, Tim Huang, Kenneth Nephew, Yunlong Liu, Lang Li

**Affiliations:** 1 Center for Computational Biology and Bioinformatics, Indiana University School of Medicine, Indianapolis, IN 46202, USA; 2 Department of Biomedical Informatics, The Ohio State University, Columbus, OH 43210, USA; 3 Laboratory of Breast Cancer Epigenomics, The Ohio State University, Columbus, OH 43210, USA; 4 Laboratory of Ovarian Cancer Epigenomics, Indiana University, Bloomington, IN 47405, USA

## Abstract

**Background:**

Typical analysis of time-series gene expression data such as clustering or graphical models cannot distinguish between early and later drug responsive gene targets in cancer cells. However, these genes would represent good candidate biomarkers.

**Results:**

We propose a new model - the dynamic time order network - to distinguish and connect early and later drug responsive gene targets. This network is constructed based on an integrated differential equation. Spline regression is applied for an accurate modeling of the time variation of gene expressions. Then a likelihood ratio test is implemented to infer the time order of any gene expression pair. One application of the model is the discovery of estrogen response biomarkers. For this purpose, we focused on genes whose responses are late when the breast cancer cells are treated with estradiol (E2).

**Conclusions:**

Our approach has been validated by successfully finding time order relations between genes of the cell cycle system. More notably, we found late response genes potentially interesting as biomarkers of E2 treatment.

## Background

Breast cancer represents a major public health issue since it comprises 22.9% of all cancers in women and it is an important cause of death [1]. Some breast cancers are sensitive to hormones such as estrogen (E2) [2]. Thus it is possible to treat these cancers by blocking the effects of these hormones, using for instance tamoxifen [3]. The discovery of biomarkers of the response to drugs is an important task in medical research because it helps know if a drug is effective for a specific patient and how it is metabolized by his organism. Biomarkers play thus an important role in personalized medicine, such as in the choice of the most relevant treatment.

Biomarkers often refer to proteins measured in the blood whose concentrations reflect the presence or the severity of the disease. In the case of estrogen treatment, biomarkers can be seen as parameters reflecting the effects of the drug on the patient. The biomarkers of hormone therapy of the breast cancer is not well developed. For instance, although tamoxifen's pharmacology mechanism is well known, its clinical biomarker is not well established yet. Understanding the cascade of estrogen signaling pathway is the key to study the potential biomarkers.

Gene expression-based biomarker discovery has demonstrated efficiency for breast cancer [4,5]. Standard methods rely on computing correlations between gene expressions and drug treatment status. Simple statistical procedures are used such as t-tests to assess the significance of over- or under-expressions of genes before and after treatment in steady-state analysis [6]. Clustering has also been successfully used for revealing particular patterns of expression [7].

Unfortunately standard methods might fail to reveal key biomarkers, since they do not take into account the temporal aspect of gene expression and the complex network of gene regulation. To tackle this issue, the analysis of time series data through dynamic networks represents efficient alternatives [8]. In this context, three main approaches can be distinguished: dynamic Bayesian networks, information-theoretic networks and ordinary differential equations. Dynamic Bayesian networks (DBNs) have been successfully applied to infer causal gene networks [9,10]. Conditional independences encoded in DBNs guarantee to infer direct relations between genes. The second approach consists in inferring the structure of dependences through an information-theoretic framework [11,12]. Most notably, the data processing inequality principle helps discard the majority of indirect dependences without involving time consuming algorithms such as those for DBNs. The last method relies on ordinary differential equations (ODEs) [13,14]. In this method, changes of gene expression are related to each other through a system of differential equations. Most notably, this method accurately and explicitly models the continuous time aspect of gene expression. Recently a combination of ODEs and DBNs has been proposed for taking into account both causal discovery (DBNs) and accurate modeling (ODEs) of gene expression [15].

Late response genes might represent relevant biomarkers because they are more stable over the time. Our approach relies on this biological aspect of biomarker discovery. To identify late response genes, we propose a new model based on a dynamic time order network (DTON). The model interpretation is simple and intuitive: it reflects which genes express in the early times and which ones in the late times after the hormone treatment. The DTON is constructed based on an integrated differential equation. Spline regression is applied for an accurate modeling of the time variation of gene expressions. A likelihood ratio test is implemented to infer the time order of any gene expression pair. The advantages of this modeling approach are numerous: (i) closed-form expressions of ODEs, (ii) accurate modeling of the time series data by using spline regression and by integrating differential equations, and (iii) model learning involving simple regressions quick to compute and only a few parameters have to be estimated. The method has been validated by successfully finding time order relations between genes of the cell cycle system. Most importantly, we found late response genes as candidate biomarkers of E2 treatment.

This paper is organized as follows. Section Materials and methods first describes experiments and data preprocessing. Late response genes are defined and discussed. Then the dynamic time order network and its model learning are presented. It is described how dynamic time order relations between genes are inferred through a likelihood ratio test. The next section illustrates our method on real data analysis. Our model is validated with the well-known cell cycle system. Late response genes of E2 treatment are discovered. Finally, the last section concludes and points out promising perspectives.

## Materials and methods

### Experiment and data preprocessing

The gene expression data come from estrogen stimulated ZR_75_1 cells. *G*_0 _- *G*_1 _synchronization cells were treated with 10^-8 ^*M *of 17 *β *- *estradiol *(E2). Then RNA was extracted from the cells before (0) or after 1, 2, 4, 6, 8, 12, 16, 20, 24, 28 and 32 hours of stimulation. For more details, the reader is referred to the original study [16]. There are 48702 probes in the original study and some of them are duplicated. Duplicated probes are averaged. Then only highly differentially expressed genes are considered through the following method. Standard deviation and mean were computed for each mRNA. A gene is considered as not differentially expressed if its standard deviation over its mean is small. At this point, we chose 0.15 as threshold. Finally, we only kept 5003 genes with high variation of their expression. The logarithmic concentration ratio (LCR) at every time point is used. Let *C_t _*denotes the concentration at time point *t *for a gene, then the LCR at time point *t *would be log $\frac{C_{t}}{C_{0}}$. The LCR indicates how much the concentration increases or decreases from the concentration at the first time point. In order to unify the variance for different genes, we standardized the LCRs at each time point.

### Late response gene

In breast cancer cells, Cicatiello *et al. *[16] showed that all the major time dependent gene expression profile clusters follow two major patterns: (i) go up or down, then stay flat; and (ii) go up or down first, stay flat, then go down or up, respectively. These patterns can be captured by a natural cubic spline function divided in three parts using two knots. The early response genes are thus defined as either up- or down-regulated genes before 5.333 hours, following E2 stimulation. The late response genes are defined as either up- or down-regulated genes after 17.333 hours. The time points 5.333 hours and 17.333 hours represent the 33th and 67th percentiles of the sampling time points.

Biologically, we favor late response genes because of their clinical implications. To check whether a drug works in human, *i.e*. inhibiting or simulating the target, one or multiple reliable biomarkers are useful to indicate the drug effects. An early response gene may not be predictive for the long term effect of the drug. It is always desirable to use a biomarker that can predict a sustainable effect of the drug. Therefore, a late response gene represents a better biomarker than an early response one. In our dataset, responsive genes after 17.333 hours following E2 treatment are likely to be the best biomarkers.

### The dynamic time order relationship

**Figure 1:**
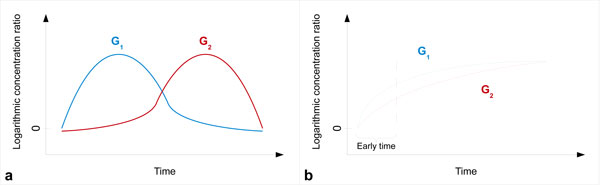
**Logarithmic concentration ratios of two genes *G*_1 _and *G*_2_**. a) A scenario showing a trivial order between the two genes. b) A more complex scenario showing an non-trivial order.

Let *f*_1_(*t*) and *f*_2_(*t*) represent the LCR curves of two genes *G*_1 _and *G*_2 _over the time *t*, as depicted in Figure 1a. Suppose *G*_1 _and *G*_2 _have a dynamic time order relation such that the expression of *G*_2 _is later than the one of *G*_1_. This relation is denoted as *G*_1 _→ *G*_2_. Then the changing rate of *G*_2 _should be related to the LCR of *G*_1 _and itself [8]. The model is an ODE:

(1) $$\frac{\text{d}f_{2}\left(t\right)}{\text{d}t}=k_{1}\;f_{1}\left(t\right)+k_{2}\;f_{2}\left(t\right).$$

In Equation (1), $\frac{\text{d}f_{2}\left(t\right)}{\text{d}t}$ represents the changing rate of *G*_2 _expression. Alternatively, Equation (1) can be expressed by integration:

(2) $$f_{2}\left(t\right)=k_{1}\;F_{1}\left(t\right)+k_{2}\;F_{2}\left(t\right).$$

In Equation (2), *F*_1_(*t*) and *F*_2_(*t*) represent the cumulative expression of *G*_1 _and *G*_2_. The integration of the ODE can help to better distinguish which gene is firstly expressed in a non-trivial scenario, such as the one presented in Figure 1b. In this example, we can see that it is possible to infer the dynamic time order relation between *G*_1 _and *G*_2 _only during the early time (because only in the early time we observe a significant difference between the two rates). By integrating the ODE (see Equation (1)), the model can take into account all the variation of the gene LCR (in early and late times). Note that this dynamic time order relation does not imply any causal relation between two genes but only indicates which one is expressed after the other.

### Natural cubic spline regression

In order to apply the integrated ODE model (Equation 2), a smooth curve is required to fit gene expression over the time. For this purpose, natural cubic spline regression (NCSR) [17] represents a good choice, since it provides a good trade-off between fit to data and model complexity. NCSR is a third-order polynomial function:

(3) $$f_{i}\left(t\right)=\beta_{0}+\beta_{1}\;t+\beta_{2}\;t^{2}+\beta_{3}\;t^{3},$$

with *f_i_*(*t*) the LCR of gene *G_i_*. Observations *y_i _*for a gene *G_i _*are regressed by the NCSR function:

(4) $$y_{i}=\beta_{0}+\beta_{1}\;t+\beta_{2}\;t^{2}+\beta_{3}\;t^{3}+\varepsilon_{SRi},$$

with $\varepsilon_{SRi}\sim N\left(0,\sigma_{SRi}^{2}\right)\cdot \varepsilon_{SRi}$and $\sigma_{SRi}^{2}$ respectively denote the residuals of the spline regression and their variance associated with the gene *G_i_*.

The time interval of our gene expression data is *t *∈ [0; 32] hours. We divide the function *f_i _*into three parts using two knots at 5.333 hours and 17.333 hours. The decomposition of the cubic function using knots is presented in Additional file 1.

Let ***β***_*ij *_= (*β*_*ij*0_, *β*_*ij*1_, *β*_*ij*2_, *β*_*ij*3_)*^T ^*and **t **= (1, *t, t*^2^, *t*^3^), then $y_{i}\sim N\left(t\beta_{ij},\sigma_{SRi}^{2}\right)$ at time *t*. The distribution of *y_i _*is written as:

(5) $$P\left(y_{i}\right)=\frac{1}{\sqrt{2\pi \sigma_{SRi}^{2}}}\text{exp}\left(-\frac{{\left(y_{i}-t\beta_{ij}\right)}^{2}}{2\sigma_{SRi}^{2}}\right),$$

with $j=1+\sum_{h=1}^{2}1\left(t>K_{h}\right)$. The value of *j *refers to the first, second or third interval.

In our study, we have 12 different time points *t *∈ {0, 1, 2, 4, 6, 8, 12, 16, 20, 24, 28, 32} and their associate LCRs for the gene *G_i _*are the vector **y***_i _*= (*y*_*i*0_, ..., *y*_*i*32_). Based on Equation 5, the likelihood for the NCSR model of gene *G_i _*for the set of 12 independently and identically distributed (i.i.d.) samples  D is:

(6) $$\begin{array}{ccc} L\left(\beta_{i1},\;\beta_{i2},\;\beta_{i3},\;\sigma_{SRi}^{2}|D\right)\; & ={\left(2\pi \sigma_{SRi}^{2}\right)}^{\frac{-12}{2}}\times \Pi_{0\leq t<K_{1}}\text{exp}\left(-\frac{{\left(y_{it}-t\beta_{i1}\right)}^{2}}{2\sigma_{SRi}^{2}}\right) & \\ & \times \Pi_{K_{1}\leq t<K_{2}}\text{exp}\left(-\frac{{\left(y_{it}-t\beta_{i2}\right)}^{2}}{2\sigma_{SRi}^{2}}\right)\times \Pi_{K_{2}\leq t\leq 32}\text{exp}\left(-\frac{{\left(y_{it}-t\beta_{i3}\right)}^{2}}{2\sigma_{SRi}^{2}}\right). \end{array}$$

The parameters ***β***_*ij *_are learned by maximizing the likelihood in Equation 6 with constrains (see Additional file 1). There are 12 parameters in the cubic function. However, only 4 out of the 12 parameters are free, as constrains must be satisfied. If we set $\beta_{i2}^{T}={\left(\beta_{i20},\beta_{i21},\beta_{i22},\beta_{i23}\right)}^{T}$ as the free parameters, we can solve parameters ***β***_*i*1 _and ***β***_*i*3 _(see Additional File 2).

We can simplify the joint likelihood in Equation 6 as follows:

(7) $$\begin{array}{c} L\left(\beta_{i1},\;\beta_{i2},\;\beta_{i3},\;\sigma_{SRi}^{2}|D\right)\;={\left(2\pi \sigma_{SRi}^{2}\right)}^{\frac{-12}{2}}\times \Pi_{0\leq t<K_{1}}\text{exp}\left(-\frac{{\left(y_{it}-t^{*}\beta_{i1}\right)}^{2}}{2\sigma_{SRi}^{2}}\right)\\ \;\;\;\;\;\;\;\;\;\times \Pi_{K_{1}\leq t<K_{2}}\;\text{exp}\left(-\frac{{\left(y_{it}-t^{*}\beta_{i2}\right)}^{2}}{2\sigma_{SRi}^{2}}\right)\times \Pi_{K_{2}\leq t\leq 32}\text{exp}\left(-\frac{{\left(y_{it}-t^{*}\beta_{i3}\right)}^{2}}{2\sigma_{SRi}^{2}}\right), \end{array}$$

where t* can be solved by the following way:

(8) $$\begin{array}{cccc} t^{*} & = & \left\{1,t,\frac{t^{3}}{3K_{1}}+K_{1}t-\frac{K_{1}^{2}}{3},t^{3}\right\}, & \left(0\leq t<K_{1}\right)\\ t^{*} & = & \left\{1,t,t^{2},t^{3}\right\}, & \left(K_{1}\leq t<K_{2}\right)\\ t^{*} & = & \left\{1,t,\frac{t^{3}-96t^{2}+3K_{2}^{2}t-3K_{2}^{2}}{3K_{2}-96},K_{2}^{3}-3K_{2}^{2}t+\frac{3K_{2}t^{3}-288K_{2}t^{2}+9K_{2}^{3}t-3K_{2}^{4}}{3K_{2}-96}\right\}, & \left(K_{2}\leq t\leq 32\right). \end{array}$$

**Figure 2:**
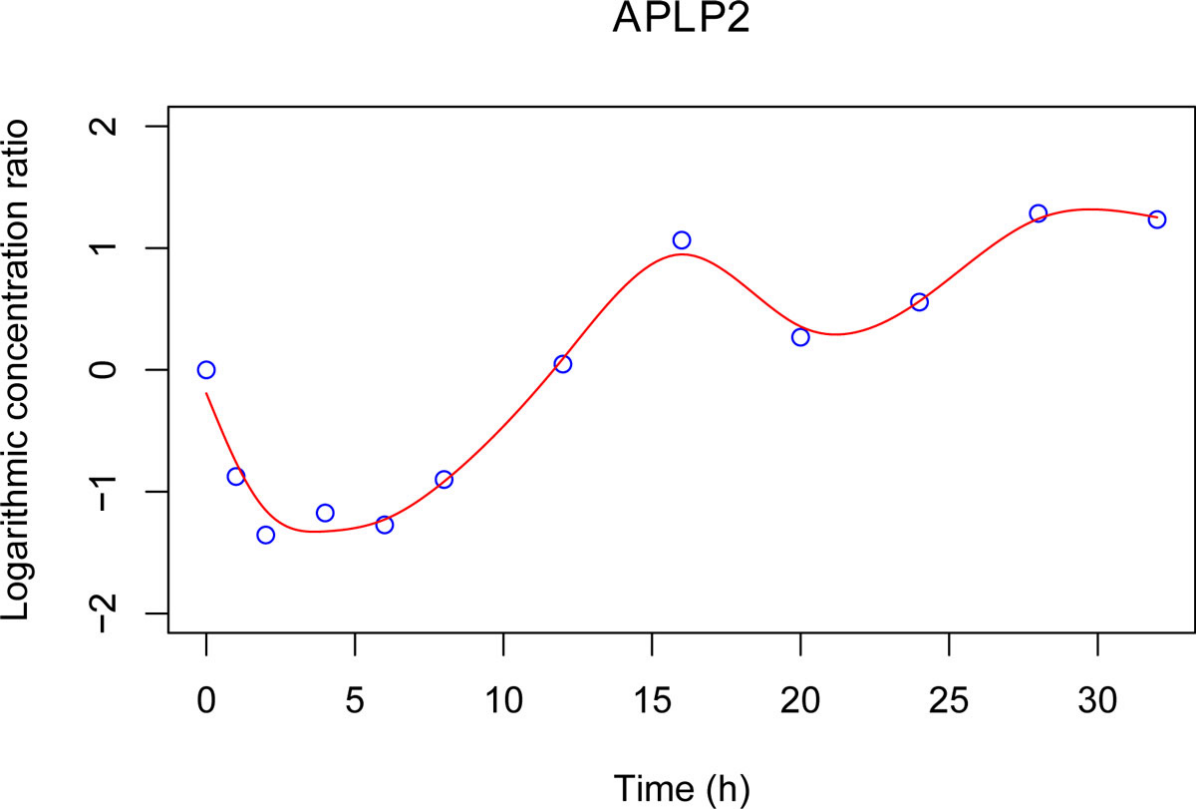
**Gene expression of APLP2 fitted by natural cubic spline regression with 2 knots *K*_1 _and *K*_2_**. *K*_1 _= 5.333 hours and *K*_2 _= 17.333 hours.

The maximum likelihood estimator of ***β***_*i*2 _for gene *G_i _*can be computed through a multiple linear regression:

(9) $${\hat{\beta }}_{i2}={\left(T^{*T}T^{*}\right)}^{-1}T^{*T}y_{i},$$

with T* a 12-by-4 matrix (presented in Additional file 3). In the matrix T*, each row *k *corresponds to the vector t* at the time point $T_{k}$ of the vector $T^{T}={\left(0,\text{ 1},\text{ 2},\text{ 4},\text{ 6},\text{ 8},\text{ 12},\text{ 16},\text{ 2}0,\text{ 24},\text{ 28},\text{ 32}\right)}^{T}$. As previously mentioned, using the parameters ${\hat{\beta }}_{i2}$, we can estimate the parameters ${\hat{\beta }}_{i1}$ and ${\hat{\beta }}_{i3}$ (see Additional file 2). Then with all these parameters, we can obtain a smooth curve to represent *f_i_*(*t*) for gene *G_i _*in the whole time interval 0 to 32 hours, as Figure 2 shows for the gene APLP2. Therefore the ODE in Equations 1 and 2 can be applied.

### Time order determination

Based on Equation 2, the dynamic time order relationship between two genes can be learned using the following multiple linear regression:

(10) $$y_{it}=b_{i0}+b_{i1}F_{1}\left(t\right)+b_{i2}F_{2}\left(t\right)+\varepsilon_{MRi},$$

with $\varepsilon_{MRi}\sim N\left(0,\;\sigma_{MRi}^{2}\right)\cdot \;\varepsilon_{MRi}$ and $\sigma_{MRi}^{2}$ respectively denote the residuals of the multiple regression and their variance associated with gene *G_i_*. The response variable **y***_it _*is the LCR of gene *G_i _*at time *t *and the predictor variables are integrations of the cubic functions at time *t*. For a predictor variable, the integration *F_i _*of piecewise cubic functions *f*_*i*1_, *f*_*i*2 _and *f*_*i*3 _is calculated as follows:

(11) $$\begin{array}{cccc} F_{i}\left(t\right) & = & \int_{0}^{t}f_{i1}\left(t\right), & \left(0\leq t<K_{1}\right)\\ F_{i}\left(t\right) & = & C_{1}+\int_{K_{1}}^{t}f_{i2}\left(t\right), & \left(K_{1}\leq t<K_{2}\right)\\ F_{i}\left(t\right) & = & C_{2}+\int_{K_{2}}^{t}f_{i3}\left(t\right), & \left(K_{2}\leq t\leq 32\right), \end{array}\;$$

where $C_{1}=\int_{0}^{K_{1}}f_{i1}\left(t\right)$ and $C_{2}=\int_{K_{1}}^{K_{2}}f_{i2}\left(t\right)+C_{1}$ are constant terms. They vary for different gene LCRs.

We apply the model in Equation 10 to every pair of genes to determine whether there is a dynamic time order relation between them. The pairwise regression models for two genes *G*_1 _and *G*_2 _are:

(12) $$y_{1}=Xb_{1}+\varepsilon_{MR1}$$

(13) $$y_{2}=Xb_{2}+\varepsilon_{MR2},$$

with $\varepsilon_{MRi}\sim N\left(0,\;\sigma_{MRi}^{2}\right)$. Vectors $y_{1}^{T}={\left(y_{10},\;\ldots ,\;y_{132}\right)}^{T}$ and $y_{2}^{T}={\left(y_{20},\;\ldots ,\;y_{232}\right)}^{T}$ are the LCRs for the pair of genes *G*_1 _and *G*_2_, and $b_{i}^{T}={\left(b_{i0},\;b_{i1},\;b_{i2}\right)}^{T}$ are the associate parameters in the model presented in Equation 10. Let *F_i_*(*t*) be the integration of *f_i_*(*t*) getting from Equation 11 and $F_{i}^{T}={\left(F_{i}\left(0\right),\;\ldots ,\;F_{i}\left(32\right)\right)}^{T}$ be the function values for *F_i_*(*t*) at each time point *t*. Then the predictor variable is **X **= (**1, F**_1_, **F**_2_).

Thus in Equations 12 and 13, values of **y***_i _*(left hand side) come from data and values of **X **(right hand side) result from the integration of the NCSR functions. For the pair of genes *G*_1 _and *G*_2_, the model in equation 12 represents the dynamic time order relation *G*_2 _→ *G*_1 _and the model in equation 13 represents the dynamic time order relation *G*_1 _→ *G*_2_.

Pairwise regressions are then computed for all pairs of genes and the log-likelihoods are calculated (see Additional file 4). In order to find whether a pair of genes has a dynamic time order relation, we look at their log-likelihood difference. If two genes present a dynamic time order relation, the regression relying on the true relation will have a better log-likelihood value than the regression based on the wrong relation, as Equations 12 and 13 represent two different dynamic time orders.

### Network construction

After determining the time order relationships, an *n*-by-*n *adjacency matrix (*n *is the number of genes) is constructed whose weights are the previously computed log-likelihood differences. In the matrix, for a couple of genes, only the positive log-likelihood difference value is kept and the negative (symmetric) log-likelihood difference value is set to 0. This adjacency matrix represents the complete directed graph of time order relationships.

#### Small network

When the network is small (less than one hundred nodes), it is interesting to keep as much as possible information about time order relations. The best strategy in this case is fine tune a threshold used to remove non-significant edges. For this purpose, a simple and efficient approach is the use of the median or other quantiles of the distribution of log-likelihood difference values. Then a simplification step is used to remove redundant edges. For instance, when one observes *A *→ *B *and *B *→ *C*, then *A *→ *C *is considered as redundant and is removed. For graph drawing, the Sugiyama's algorithm [18] provides a hierarchical display which is particularly relevant for reflecting time order relations.

#### Genome-wide network

When the network is huge, such as the genome-wide network from the microarray data, the previous approach cannot be used. The reason is that a low threshold value will create a network highly connected which is too complex to manipulate and to visualize, whereas a high threshold value will lead to a graph with many connected components from which it will only be possible to infer time orders between connected genes. To tackle this issue, we compute the so-called maximum weight spanning tree (MWST). This graph presents several advantages: (i) its tree shape is a very simple structure easy to manipulate and visualize, and (ii) every node is connected by a path such that we can access to the time order relation between each gene. Besides, the MWST can be quickly computed in *O*(*n*^2^*logn*) through the Prim's algorithm [19]. Regarding graph drawing, the Sugiyama's algorithm cannot be used when the graph is too huge. Instead we prefer to display it using an algorithm specific to tree drawing, the Bubble tree algorithm [20].

### Biological interpretation of the model

**Figure 3:**
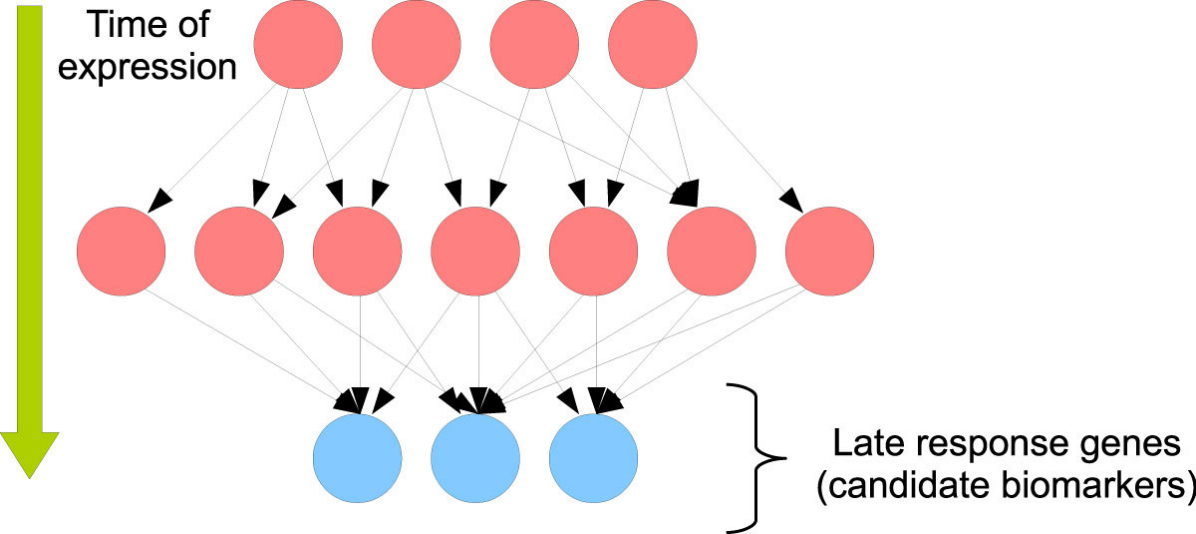
**Dynamic time order network and its biological interpretation**. The blue nodes indicate the late response genes whereas the red nodes point out the remaining genes.

The dynamic time order network (DTON) has a biological interpretation. It is illustrated in Figure 3. In this network, late response genes are hubs which are connected by many incoming pathways. Thus the identification of these hubs helps find candidates for biomarkers of breast cancers. Based on this idea, we propose a criterion to identify late response genes in the network. Late response genes are defined as nodes only connected to incoming edges, and the more incoming edges a node has the later is considered its response. We call these nodes "incoming-edge hubs".

### Implementation

Our learning method is implemented in R. The R source code is available on request. For graph drawing and display, the software Tulip (http://tulip.labri.fr/TulipDrupal/) was used. It is a user-friendly tool able to deal with about one million nodes.

## Results and discussion

### Reproducing the cell cycle temporal system

**Figure 4:**
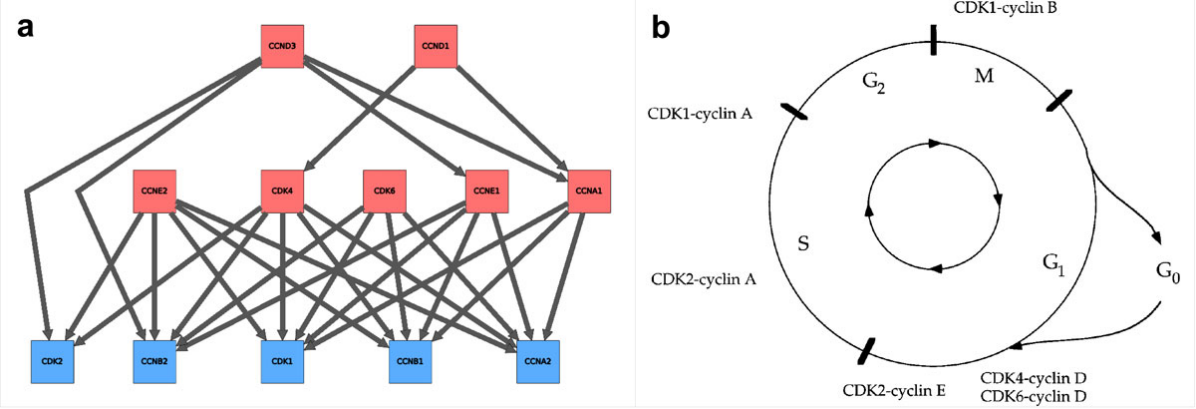
**Cell cycle temporal system modeling**. a) The inferred dynamic time order network. b) A schematic representation of the cell cycle temporal system [26]. The color code is the same as in Figure 3.

The cell cycle temporal system represents a good benchmark for evaluating our method. In this subsection, in order to see if we can reproduce the time order relations, we focused on key cell cycle genes. Twelve mRNA expression data were selected, which include cyclin A1 (CCNA1), cyclin A2 (CCNA2), cyclin B1 (CCNB1), cyclin B2 (CCNB2), cyclin D1 (CCND1), cyclin D3 (CCND3), cyclin E1 (CCNE1), cyclin E2 (CCNE2), cyclin-dependent kinase 1 (CDK1), cyclin-dependent kinase 2 (CDK2), cyclin-dependent kinase 4 (CDK4) and cyclin-dependent kinase 6 (CDK6). Regressions have be computed for all pairs of genes. Then, the network of cell cycle genes has been computed by thresholding using the median of the log-likelihood differences. After simplification, the inferred network is composed of 27 time order relations. It is depicted in Figure 4a. The reference network of cell cycle genes is displayed in Figure 4b. Over the 27 time order relations inferred, 24 correspond to the reference network, 0 are wrong and 3 cannot be checked from the reference network (because the reference network is not enough accurate). The network is thus recovered with at least 89% of accuracy. More notably, the network points out 5 incoming-edge hubs: CDK2, CCNB2, CDK1, CCNB1 and CCNA2 (these nodes are colored in blue in Figure 4a). The genes CCNB2, CDK1, CCNB1 and CCNA2 correspond to late response genes in the reference network. Regarding CDK2, it should be considered as an intermediate response gene. Compared to the other hubs which all show 5 incoming edges, CDK2 only presents 3 incoming edges.

### Genome-wide network

**Figure 5:**
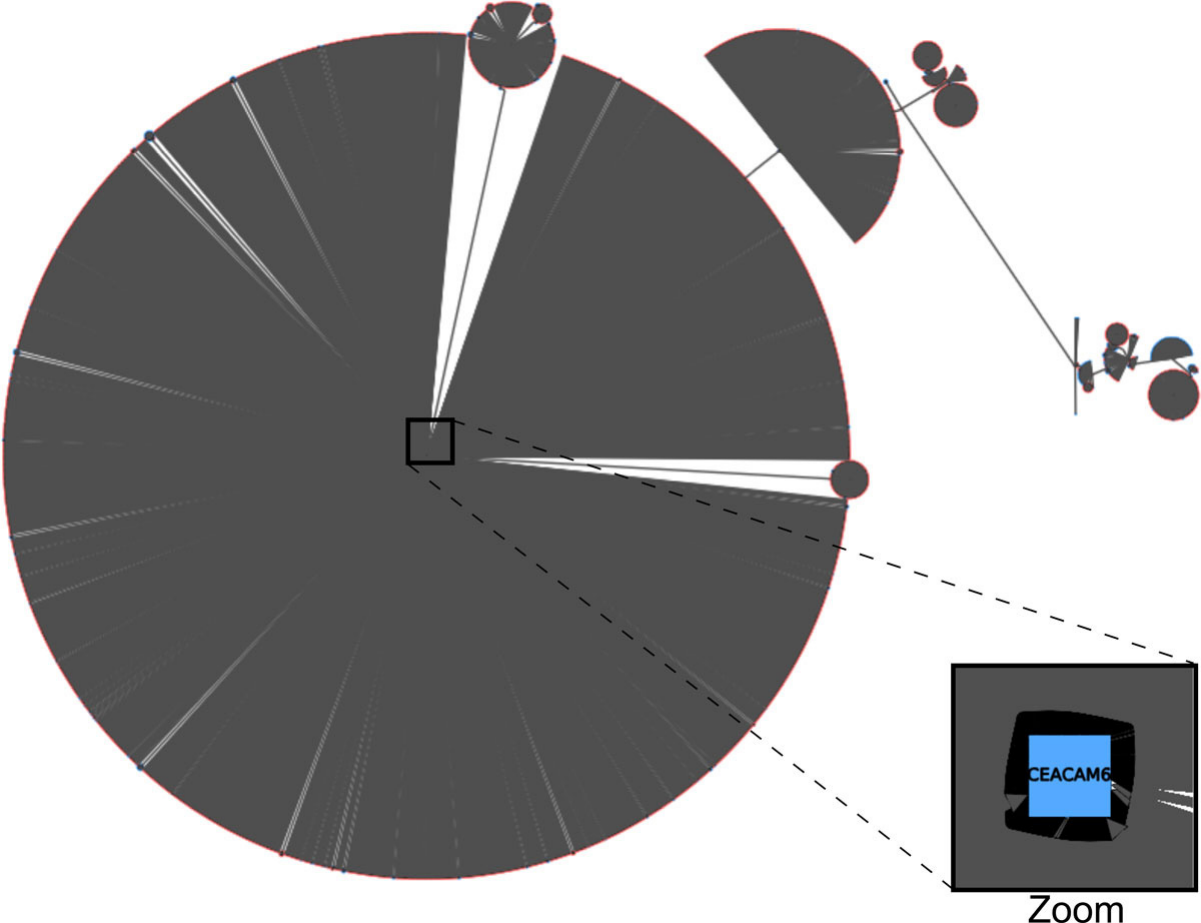
**Genome-wide dynamic time order network**. It has been computed for all the 5003 genes. Big circles represent incoming-edge hubs at the center (blue) connected to a very large number of nodes (red). The color code is the same as in Figure 3.

**Figure 6:**
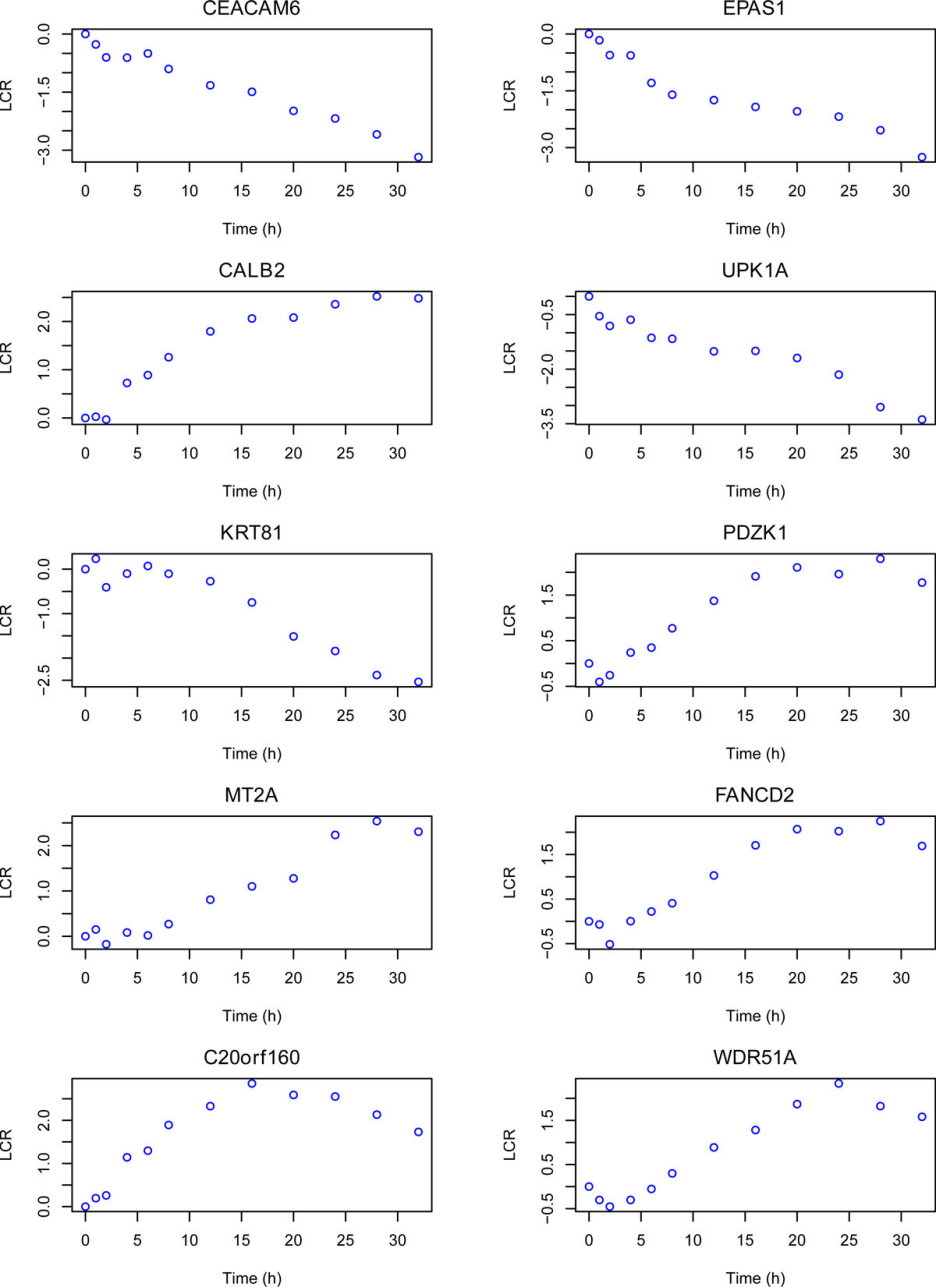
**Gene expression profiles for the 10 most important incoming-edge hubs**. LCR: logarithmic concentration ratio.

For genome-wide network modeling, an MWST has been constructed from all pairwise regressions on the 5003 genes. The network is depicted in Figure 5. We observe that this network is composed of several large incoming-edge hubs and reflects a star shape topology. The 10 most important hubs are listed in Table 1. We observe that CELCAM6 is connected to 2783 incoming edges (CELCAM6 is magnified in the Figure 5). Other large hubs are EPAS1, CALB2, UPK1A, KRT81, PDZK1, MT2A, FANCD2, C20orf160 and WDR51A, in the decreasing order of importance. The profiles of expression over time are presented in Figure 6. All these profiles reflect a late under- or overexpressed response. CELCAM6, EPAS1, UPK1A and KRT81 are genes whose expressions decrease over the time, whereas CALB2, PDZK1, MT2A, FANCD2, C20orf160 and WDR51A are overexpressed after E2 treatment.

**Table 1:** List of the 10 most important incoming-edge hubs.

Gene	Number of incoming edges	Expression
CEACAM6	2783	Underexpressed

EPAS1	417	Underexpressed

CALB2	250	Overexpressed

UPK1A	171	Underexpressed

KRT81	150	Underexpressed

PDZK1	130	Overexpressed

MT2A	102	Overexpressed

FANCD2	78	Overexpressed

C20orf160	62	Overexpressed

WDR51A	49	Overexpressed

**Figure 7:**
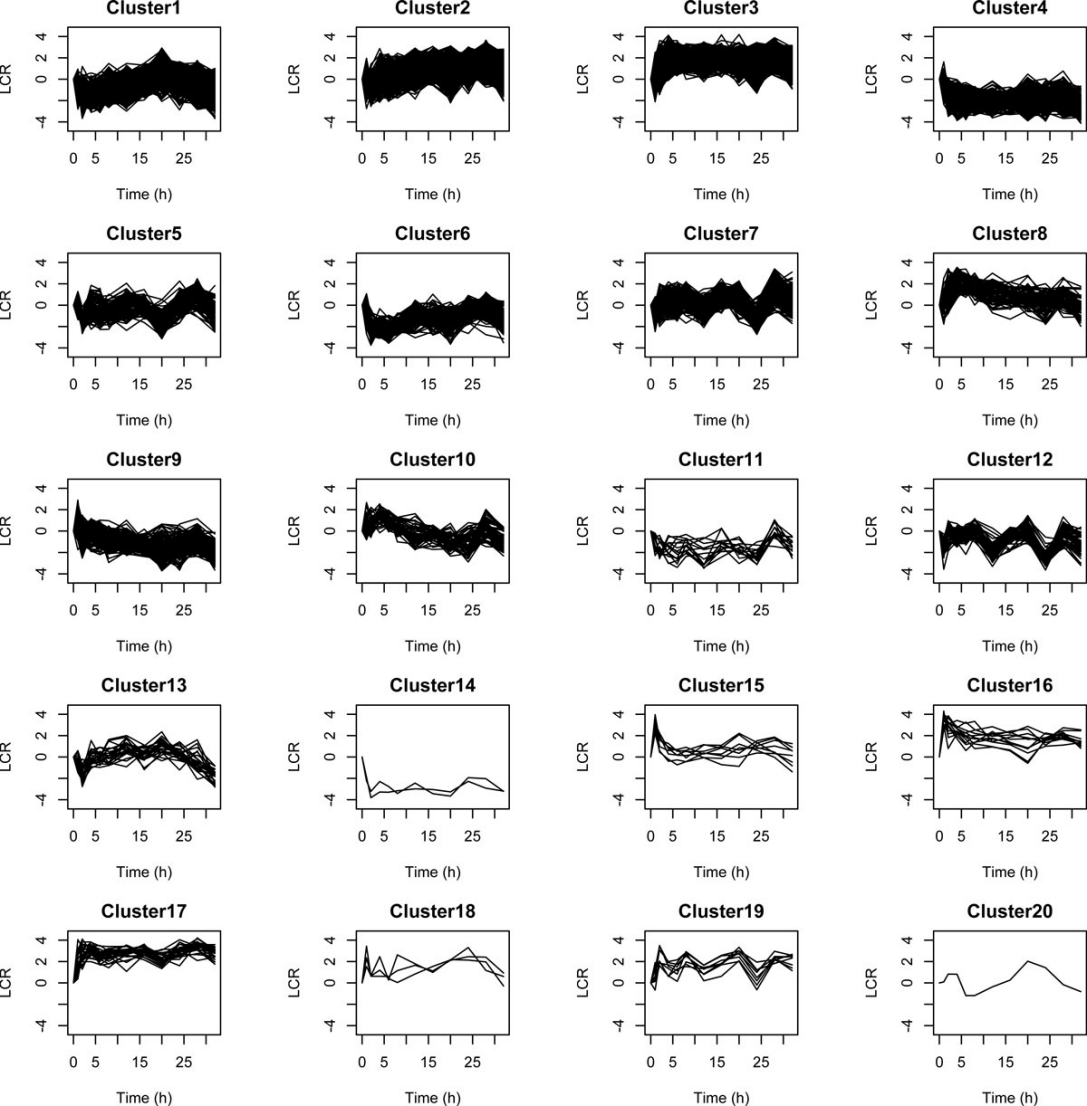
**Cluster profiles obtained with agglomerative hierarchical clustering, for all the 5003 genes**.

**Figure 8:**
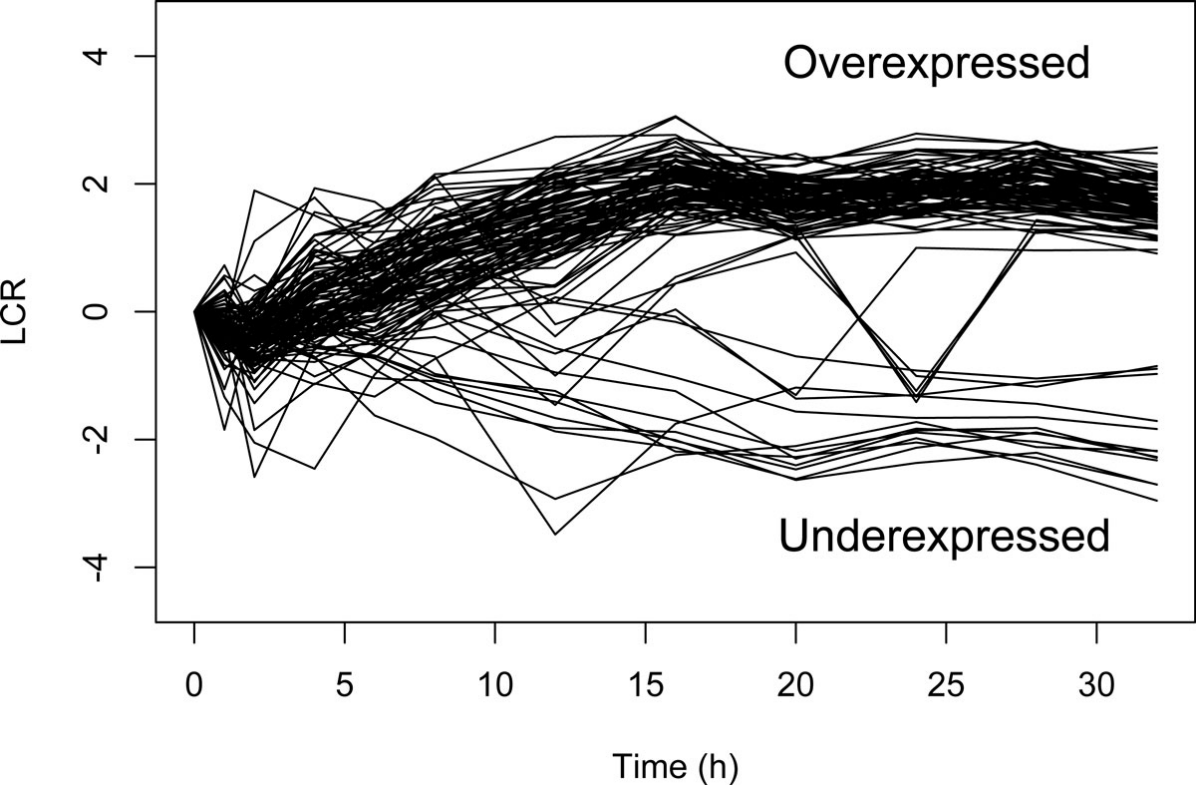
**Profiles of late response genes identified using t-tests**.

**Figure 9:**
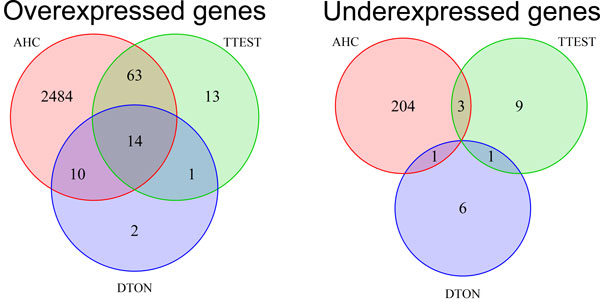
**Venn diagram to compare the late response genes identified from the dynamic time order network (DTON), the agglomerative hierarchical clustering (AHC) and the t-tests**.

The identification of late response genes does not represent a well-studied issue. Most notably, no dedicated method has been developed for this purpose. Nevertheless, we tried to compare our method with standard approaches in gene expression analysis: agglomerative hierarchical clustering (AHC) and t-tests. On the one hand, AHC is a well-used tool to cluster gene expression profiles. After computing AHC, we used the silhouette criteria to determine the optimum number *k *of clusters [21]. We obtain the best silhouette values for *k *= 5. However, when we looked at the clusters, we were unable to identify any cluster corresponding to late response genes. We thus tried with higher values of *k*. With *k *= 20, we are able to more accurately distinguish different trends in gene expression (see Figure 7). However, it is still hard to identify late response genes. The clusters 2 and 9 might represent candidates for over-expressed and under-expressed late response genes, respectively. On the other hand, we used a t-test strategy. We obtained better results. Our strategy was the following: (i) first we selected genes whose deviations of absolute LCR values from 0 for the first time points 0, 1, 2, 4, 6, 8, 12 and 16 are non-significant (*p *- *value *>*quantile*(0.6)), and (ii) then, from these selected genes, we only kept those whose absolute LCR values for the last time points 20, 24, 28 and 32 are significantly different from 0 (*p *- *value *<*quantile*(0.05)). Profiles of selected genes are depicted in Figure 8. With t-tests we observe a better identification of late response genes than with AHC. However we notice that some of these gene expressions oscillate between over- and under-expression for the last time points. Figure 9 shows the Venn diagrams for the comparison of results between DTON (our method), the AHC and the t-test strategy. The t-test strategy and DTON are both very specific with a few number of genes identified: 91 and 27 over-expressed genes, and 13 and 8 under-expressed genes for t-tests and DTON, respectively. For over-expressed genes, more than half of the genes found with DTON are also identified with t-tests. Regarding under-expressed genes, few genes are shared. Comparatively, AHC is much less specific with around 2600 over-expressed and around 200 under-expressed genes. It is thus not surprising that AHC shares a large proportion of over-expressed genes with DTON and t-tests.

We also search in the literature if the late response genes identified with our method can be good candidate biomarkers. Since biomarkers are molecules that are observed in cancer patients but not in healthly people, there are likely to be genes overexpressed after E2 treatment. Among the overexpressed hubs of the network, CALB2, PDZK1, MT2A and FANCD2 are well-known in the literature as diagnostic marker of breast cancer and E2 response [22-25]. Besides, C20orf160 is reported in the Genes-to-Systems Breast Cancer Database (http://www.itb.cnr.it/breastcancer//index.html). WDR51A (also called POC1A) is found associated with breast cancer in The Human Protein Atlas (http://www.proteinatlas.org/).

## Conclusion

Based on experimentations carried out on time-series gene expression data, our dynamic time order network has been shown to efficiently distinguish and connect early and late response genes. First, our model has faithfully reproduced the cell cycle temporal system. Over the 27 time order relations inferred, 89% correspond to the state-of-art network, 11% cannot be checked, but no one are false. Second, our approach has been successfully applied to a genome-wide level. The learning method has been able to process five thousands genes and the network simplification through the maximum weighted spanning tree provided a graphical display of the huge network. Most notably, several incoming-edge hubs showing very high connectivity have been discovered. All these hubs showed late gene response profiles. Regarding those which are overexpressed over the time, they have been reported as biomarkers of breast cancer and E2 response in the literature and databases.

The comparison of results with other approaches is not straightforward, since our method is the only one dedicated to identify late response genes. When compared with standard methods in gene expression analysis, our approach yielded specific results, contrary to agglomerative hierarchical clustering. Moreover it does not need any complex thresholding such as with a t-test strategy. It is worth noting that all genes identified with DTON showed late responses, while this is not the case with the t-test strategy. Besides, our approach is based on the comparison of gene expression integrals combined with cubic spline regression, thus offering an accurate assessment of time order relations.

The discovery of biomarkers is one of the application of our model. The distinction between early and late response genes is also an important application in developmental biology where the understanding of the temporal aspect of gene expression is a key issue such as for cell differentiation. For the moment, we mainly focused on the identification of late response genes. The use of another graph modeling would be more efficient for pointing out early response genes than the MWST which tends to display incoming-edge hubs.

## Competing interests

The authors declare that they have no competing interests.

## Authors' contributions

PZ and RM both wrote the paper. PZ, RM, YX, KH and LL conceived the dynamic time order network. PZ and RM carried out the implementation and the experiments. LL, TH, KN and YL designed the study and participated in its coordination. All authors read and approved the final version of the manuscript.

## Supplementary Material

Additional file 1 **Decomposition of the cubic function using knots**. Click here for file

Additional file 2 **Solving of parameters *β*_*i*1 _and *β*_*i*3_**. Click here for file

Additional file 3 Matrix T*. Click here for file

Additional file 4 **Likelihood computation of regression for the time order determination**. Click here for file
