# Supplementary material for: A dynamic time order network for time-series gene expression data analysis
Source: BMC Syst Biol. 2012 Dec 17;6(Suppl 3):S9. doi: 10.1186/1752-0509-6-S3-S9 (PMC3524318; doi:10.1186/1752-0509-6-S3-S9)
Supplement: Additional file 1 — Decomposition of the cubic function using knots. [file 1752-0509-6-S3-S9-S1.pdf]

Decomposition of the cubic function using knots

The two knots are respectively denoted K_1 and K_2, with $K_1 = 5.333\ h$ and $K_2 = 17.333\ h$. Therefore the function $f_i(t)$ is composed of three cubic functions $f_{i1}(t)$, $f_{i2}(t)$ and $f_{i3}(t)$ for a gene G_i in the whole time interval:

$$f_{i1}(t) = \beta_{i10} + \beta_{i11}\ t + \beta_{i12}\ t^2 + \beta_{i13}\ t^3, \quad (0 \le t < K_1)$$

$$f_{i2}(t) = \beta_{i20} + \beta_{i21}\ t + \beta_{i22}\ t^2 + \beta_{i23}\ t^3, \quad (K_1 \le t < K_2)$$

$$f_{i3}(t) = \beta_{i30} + \beta_{i31}\ t + \beta_{i32}\ t^2 + \beta_{i33}\ t^3, \quad (K_2 \le t \le 32).$$

The three functions $f_{i1}(t)$, $f_{i2}(t)$ and $f_{i3}(t)$ should satisfy the conditions such that themselves, their first and second derivatives are continuous at each knot. Also their second derivatives are null at times 0 h and 32 h. These constrains can be written as:

$$f_{i1}(K_1) = f_{i2}(K_1), \quad \frac{\mathrm{d}f_{i1}(K_1)}{\mathrm{d}t} = \frac{\mathrm{d}f_{i2}(K_1)}{\mathrm{d}t}, \quad \frac{\mathrm{d}^2 f_{i1}(K_1)}{\mathrm{d}t^2} = \frac{\mathrm{d}^2 f_{i2}(K_1)}{\mathrm{d}t^2}, \quad \frac{\mathrm{d}^2 f_{i1}(0)}{\mathrm{d}t^2} = 0,$$

$$f_{i2}(K_2) = f_{i3}(K_2), \quad \frac{\mathrm{d}f_{i2}(K_2)}{\mathrm{d}t} = \frac{\mathrm{d}f_{i3}(K_2)}{\mathrm{d}t}, \quad \frac{\mathrm{d}^2 f_{i2}(K_2)}{\mathrm{d}t^2} = \frac{\mathrm{d}^2 f_{i3}(K_2)}{\mathrm{d}t^2}, \quad \frac{\mathrm{d}^2 f_{i3}(32)}{\mathrm{d}t^2} = 0.$$
