# Supplementary material for: A dynamic time order network for time-series gene expression data analysis
Source: BMC Syst Biol. 2012 Dec 17;6(Suppl 3):S9. doi: 10.1186/1752-0509-6-S3-S9 (PMC3524318; doi:10.1186/1752-0509-6-S3-S9)
Supplement: Additional file 2 — Solving of parameters βi1 and βi3. [file 1752-0509-6-S3-S9-S2.pdf]

Solving of parameters $\boldsymbol{\beta}_{i1}$ and $\boldsymbol{\beta}_{i3}$.

For parameters $\boldsymbol{\beta}_{i1}$, constrains in Additional file 1 can be written as:

$$\beta_{i10} + \beta_{i11}K_1 + \beta_{i12}K_1^2 + \beta_{i13}K_1^3 = \beta_{i20} + \beta_{i21}K_1 + \beta_{i22}K_1^2 + \beta_{i23}K_1^3$$

$$\beta_{i11} + 2\beta_{i12}K_1 + 3\beta_{i13}K_1^2 = \beta_{i21} + 2\beta_{i22}K_1 + 3\beta_{i23}K_1^2$$

$$2\beta_{i12} + 6\beta_{i13}K_1 = 2\beta_{i22} + 6\beta_{i23}K_1$$

$$\beta_{i12} = 0$$

Solving these equations simultaneously leads to:

$$\begin{aligned}
\beta_{i10} &= \beta_{i20} - \frac{K_1^2}{3}\beta_{i22} \\
\beta_{i11} &= \beta_{i21} + K_1\beta_{i22} \\
\beta_{i12} &= 0 \\
\beta_{i13} &= \frac{\beta_{i22} + 3K_1\beta_{i23}}{3K_1}.
\end{aligned}$$

For parameters $\boldsymbol{\beta}_{i3}$, constrains in Additional file 1 can be written as:

$$\beta_{i20} + \beta_{i21}K_2 + \beta_{i22}K_2^2 + \beta_{i23}K_2^3 = \beta_{i30} + \beta_{i31}K_2 + \beta_{i32}K_2^2 + \beta_{i33}K_2^3$$

$$\beta_{i21} + 2\beta_{i22}K_2 + 3\beta_{i23}K_2^2 = \beta_{i31} + 2\beta_{i32}K_2 + 3\beta_{i33}K_2^2$$

$$2\beta_{i22} + 6\beta_{i23}K_2 = 2\beta_{i32} + 6\beta_{i33}K_2$$

$$2\beta_{i32} + 6\beta_{i33}32 = 0$$

Solving these equations simultaneously leads to:

$$\begin{aligned}
\beta_{i30} &= \beta_{i20} + K_2^3\beta_{i23} - K_2^3\frac{\beta_{i22} + 3K_2^2\beta_{i23}}{3K_2 - 96} \\
\beta_{i31} &= \beta_{i21} - 3K_2^2\beta_{i23} + 3K_2^2\frac{\beta_{i22} + 3K_2^2\beta_{i23}}{3K_2 - 96} \\
\beta_{i32} &= -96 \times \frac{\beta_{i22} + 3K_2^2\beta_{i23}}{3K_2 - 96} \\
\beta_{i33} &= \frac{\beta_{i22} + 3K_2^2\beta_{i23}}{3K_2 - 96}.
\end{aligned}$$
