# Supplementary material for: A dynamic time order network for time-series gene expression data analysis
Source: BMC Syst Biol. 2012 Dec 17;6(Suppl 3):S9. doi: 10.1186/1752-0509-6-S3-S9 (PMC3524318; doi:10.1186/1752-0509-6-S3-S9)
Supplement: Additional file 3 — Matrix T*. [file 1752-0509-6-S3-S9-S3.pdf]

Matrix $\mathbf{T}^*$.

$$\begin{pmatrix}
1 & 0 & 0 & 0 \\
1 & 1 & 1 & 1 \\
1 & 2 & 4 & 8 \\
1 & 4 & 16 & 64 \\
1 & 6 & 36 & 216 \\
1 & 8 & 64 & 512 \\
1 & 12 & 144 & 1728 \\
1 & 16 & 256 & 4096 \\
1 & 20 & 400 & 8000 \\
1 & 24 & 576 & 13824 \\
1 & 28 & 784 & 21952 \\
1 & 32 & 1024 & 32768
\end{pmatrix}$$
