# Supplementary material for: A dynamic time order network for time-series gene expression data analysis
Source: BMC Syst Biol. 2012 Dec 17;6(Suppl 3):S9. doi: 10.1186/1752-0509-6-S3-S9 (PMC3524318; doi:10.1186/1752-0509-6-S3-S9)
Supplement: Additional file 4 — Likelihood computation of regression for the time order determination. [file 1752-0509-6-S3-S9-S4.pdf]

Likelihood computation of regression for the time order determination

Based on the normality assumption, we have $\mathbf{y}_{it} \sim \mathcal{N}(\mathbf{X}_t\mathbf{b}_i, \sigma^2_{MRi})$, $\mathbf{X}_t$ is the row of $\mathbf{X}$ at time t. Therefore the likelihood of the models in Equations 12 and 13 for the set of 12 i.i.d. samples $\mathcal{D}$ is:

$$\mathcal{L}(\mathbf{b}_i, \sigma^2_{MRi}|\mathcal{D}) = (2\pi\sigma^2_{MRi})^{\frac{-12}{2}} \Pi_{0\leq t\leq 32}\ \exp\left(-\frac{(\mathbf{y}_{it} - \mathbf{X}_t\mathbf{b}_i)^2}{2\sigma^2_{MRi}}\right).$$

The corresponding log-likelihood is:

$$\log \mathcal{L}(\mathbf{b}_i, \sigma^2_{MRi}|\mathcal{D}) = -\sum_{0\leq t\leq 32}\left(\frac{(\mathbf{y}_{it} - \mathbf{X}_t\mathbf{b}_i)^2}{2\sigma^2_{MRi}}\right) - \frac{12}{2}\log(\sigma^2_{MRi}) - \frac{12}{2}\log(2\pi).$$

Parameters $\mathbf{b}_i$ and σ^2_{MRi} are estimated by ordinary least squares:

$$\hat{\mathbf{b}}_i = (\mathbf{X}^T\mathbf{X})^{-1}\mathbf{X}^T\mathbf{y}_i$$
$$\hat{\sigma}^2_{MRi} = \frac{(\mathbf{y}_i - \mathbf{X}\hat{\mathbf{b}}_i)^T(\mathbf{y}_i - \mathbf{X}\hat{\mathbf{b}}_i)}{12}.$$

Using the estimated parameters $\hat{\mathbf{b}}_i$ and $\hat{\sigma}^2_{MRi}$, we can formulate the log-likelihood such as:

$$\log \mathcal{L}(\mathbf{b}_i, \sigma^2_{MRi}|\mathcal{D}) = -\frac{12}{2}\log\left(\frac{(\mathbf{y}_i - \mathbf{X}\hat{\mathbf{b}}_i)^T(\mathbf{y}_i - \mathbf{X}\hat{\mathbf{b}}_i)}{12}\right) + C,$$

where C is a constant term and is the same for every gene.
